# External Use of Chloroform

**Published:** 1852-06

**Authors:** 


*External Use of Chloroform.*—Dr. Channing read the following paper on this subject before the Boston Society for Medical Improvement: I have made some trials of the external application of chloroform. It has often been entirely successful in the present relief of pain. In some, the relief has been permanent. This use of the remedy of pain is not new. It has been said that a free application of chloroform to a part on which a surgical operation is to be done, will render such part insensible to the violence done it. Thus immersing a finger in chloroform will prevent the suffering which accompanies the deep cuts which some of the diseases of this member require, and which are described as exquisitely acute. These statements rest on good authority. In such use of chloroform consciousness remains unimpaired, the brain not being at all affected by it.

Having frequently applied chloroform externally for the relief of pain, in many and various cases, and often with speedy, and generally with continued relief, I propose to present some of them to the Society:—

Case I. 1848. Mrs. ——, about 30, has children. For some years has had symptoms of uterine disturbance. Among these, have been suffering on intercourse, leucorrhea, tumoral pains in and about the pelvis, always increased by walking, and which, except pain in the spine, are relieved by lying down. For the uterine conditions the usual means were faithfully employed, and her complaints there were entirely and permanently removed. The dorsal trouble, however, was as great as ever.

For this I recommended the external application of chloroform over the whole course of the spinewhich was involved in the disease. The effect was immediate and complete. I saw this patient a few days since, November, 1851, and found her well in regard to the back, and speaking of herself as very well in all respects.

In this case, the pain in the spine was doubtless the result of the reflex function, the womb being the original seat of the maladay. But when the uterine affection was relieved, the sympathetic dorsal difficulty remained, and only yielded to the use of chloroform. The quantity used was an ounce.

Case II. Mr. ——, aged 22. A very feeble man in appearance; very thin in flesh; pale; and an almost constant sufferer from headache. This pain occupied but a small part of the head arc, and most frequently the right temporal region, It was accompanied by constant nausea, frequent vomiting, great prostration of strength, and demanding for its present relief firm pressure over the part affected.

The last attack, which happened a few weeks since, I applied chloroform to the seat of pain. A few drops were applied by means of a handkerchief. The immediate effect was redness of the skin and a tinging sensation in the spot. The pain was at once relieved. He raised his head from a friend's shoulder, opened his eyes, looked round, and expressed his utter surprise and deep pleasure at the entire relief which had followed the use of such apparently simple means. He called on me the next morning, and reported himself perfectly well. I have not been called to him since.

The relief in this was *sudden* in its occurrence, as it had been in others. It has not been less permanent on that account, or in such cases. It has acted at once to remove pain, and with a rapidity which distinguishes the relief entirely from that which follows etherization by inhalation. The brain does not seem to have anything to do with the matter. Local pain is abolished, and at once; and the nerves have no story of suffering to tell to the brain. The sentinel is at his post, but his function is not needed. The part does not lose its natural sensibility at all. On the contrary, it feels the tingling, the irritation of the chloroform, just as distinctly as at first. Nothing has been lost of natural power and healthful function. All that has happened is this; the pain has ceased, and the patient is well.

Case III. 1851. Mrs. ——, aged 36. Has long suffered from uterine disturbance. The most exhausting and annoying symptom was leucorrhea. This was a perpetual drain, and made her exceedingly uncomfortable. I was occasionally consulted in this case; but, as her general health continued tolerably firm, no systematic attempt at recovery was thought necessary by the patient. She became pregnant, and supposed herself between two and three moths advanced in that state, when hemorrhage and severe uterine pain occurred, on account of which I was desired to see her. She had lost a great deal of blood before I saw her. I found her perfectly blanched—lips and whole face; skin cold; hands bloodless; faint; almost pulseless. Various means were used, and the hemorrhage was stopped. I considered her case, however, so pressing, that I passed the whole day in the house. She now began to complain of pain in the back. This had exacerbations in the night, becoming too severe to allow sleep, and continuing three or four hours without mitigation. For this I recommended chloroform, which was applied by means of a handkerchief, and with excellent results. After three applications, on as many days or nights, the pain ceased, and has not now, at the end of some weeks, returned.

Case IV. Mrs. ——, about 40. Last child, thirteen years old. I was called in consultation in this case, because of vomiting, attending pregnancy, of about three months' standing. The vomiting ceased under treatment, and food was taken in sufficient quantities, and was well borne by the stomach. A comatose state supervened, which increased, with entire abolition of mind, and death was its consequence. The termination of cases of fatal vomiting by coma has occurred in many instances which have come under my notice or knowledge. I do not, however, recollect a case in which apparent recovery has been followed by coma ending in death.

This case is reported here, because of the use of chloroform for one of its symptoms. This was pain in the back, in the course of the spine. It was very severe, taking the lead, in distress, of all the rest. For this pain I prescribed an ointment, if so liquid a substance as was produced by the union of lard and chloroform deserves the name. It was applied twice or thrice a day, and with most excellent effects, the pain entirely disappearing. It was used in the same way in the first of these cases.

Case V. Mrs. ——. Has had four premature labors; the first at about five months, the fourth at about the eighth. They were all still-born, or neither of them lived over an hour or two. The fifth pregnancy was terminated at the full time by the birth of a living, healthful male child, on the 17th October, 1851. I visited her for the usual time after delivery, and left her in rapid progress towards perfect recovery. I was called to see her on the 27th October, and found her apparently very ill. Pulse rapid; skin hot; face flushed; much pain in head and both breasts, making all attempts at nursing absolutely agonizing. A thick eruption had appeared over the whole chest. Upon examination, the breasts were found exquisitely sensitive, not bearing pressure at all. Sufficient pressure, however, was made to enable me to ascertain that there was no unusual hardness, or, in short, any other sign of disease in either organ. The pain was confined to the skin, and seemed to involve its whole tissue. The eruption proved to be a very full crop of sudamina of unusual size, resembling exactly an exaggerated form of the same kind of eruption in typhoid fever. Various means had been used before my visit, but without any good effect.

I recommended chloroform, and with the best effects. I regret I had not used it in combination with lard or rose ointment, both of which I have used, and for the reason that it may in this way be more extensively applied, with more continuous benefit, and with less of the unpleasant tingling which so generally accompanies the uncombined substance.

On what did this aching pain depend? It resembled that which at times makes *shingles* so very distressing. Was it owing to the eruption? I have never known the skin in sudamina to be complained of as the seat of pain, and I have examined it when covered by this eruption after a manner to have produced pain had morbid sensibility existed. In Mrs. ——'s case, the pain was persistent, being much aggravated by handling, though never so gently. The pain in this case subsided, under the use of chloroform, long before the eruption disappeared.

Case VI. Mrs. ——, aged 25, married very young, and had a premature labor within a year or two afterwards. I was desired to see her, October, 1851, on account of an enlargement of the abdomen, on the left side, and for various signs of ill health. The tumor was found to be an excessively enlarged spleen. It filled the left hypochondrium, extending forward to the epigastrium, and downward to Poupart's ligament. Its anterior boundary was the linea alba. From this point or line it extended laterally and backward, filling accurately one whole side of the abdomen. She has been always regular in regard to the catamenial function, with the exception of the last two periods. Nausea, vomiting, and other unusual and disagreeable feelings have led to a suspicion that pregnancy may exist. She has recently taken a fatiguing journey of three days, and has suffered much since. The pain has been most troublesome in the head, and in the seat of the tumor. Diarrhea and flatulence, with general anasarca, involving the face and head, are also present. Chloroform to the forehead and to the seat of the tumor has given her much comfort, more than has been derived from any other means used.—*American Jour. Med. Sci.*

